# Effectiveness of Anti-Cluster of Differentiation 20 as a Disease-Modifying Therapy in Multiple Sclerosis Across Its Different Phenotypes at the University Hospital of Caen

**DOI:** 10.7759/cureus.22120

**Published:** 2022-02-11

**Authors:** Moayyad S Bauthman

**Affiliations:** 1 Internal Medicine, Faculty of Medicine, King Abdulaziz University, Rabigh, SAU

**Keywords:** disease-modifying therapy, quality of life, multiple sclerosis phenotypes, anti cd-20, multiple sclerosis

## Abstract

Background

Multiple sclerosis is a chronic, demyelinating disorder occurring primarily as two main forms of relapsing-remitting multiple sclerosis (RRMS) found predominantly in women, and primary progressive multiple sclerosis (PPMS) occurring predominantly in men. In this retrospective single-center study, we aimed to explore the effects of anti-cluster of differentiation (CD)20 treatment for both relapsing-remitting and primary progressive forms of multiple sclerosis (MS) in a population-based cohort treated at the university hospital.

Methodology

The diagnostic factors being assessed were forms of multiple sclerosis (MS), age at first relapse, whereas therapeutic factors were age at first disease-modifying therapy (DMT), age at starting anti-CD20, reason to switch to anti-CD20 and the duration of anti-CD20 treatment. Primary outcomes measured were number of relapses and progression in disability as measured by the Expanded Disability Status Scale, while secondary outcomes measures being assessed number of cerebral lesions on MRI and level of IgG at the beginning and end of the 12-month treatment.

Results

Treatment with anti-CD20 demonstrated a reduction in number of relapses 12 months after treatment, no change in the progression of disability in RRMS type, but an increase in PPMS type. No change was observed in cerebral MRI lesions at the end of treatment after 12 months. A statistically significant reduction in serum IgG value was observed after 12 months from the start of treatment, where only one out of 26 (3.8%) patients developed hypogammaglobulinemia with IgG less than 6 g/L but none developed hypogammaglobulinemia of less than 5 g/L.

Conclusion

Anti-CD20 antibodies as disease-modifying therapy can profoundly impact the course and progression of MS in both its forms if utilized at an earlier stage in patients and therefore greatly improve the quality of life in patients living with multiple sclerosis.

## Introduction

Multiple sclerosis (MS) is a chronic, demyelinating inflammatory disease of the central nervous system. In France, the crude prevalence rate of MS was 151.2 per 100 000 inhabitants in 2012 [1]. It is one of the most common causes of physical disability in young adults and mainly affects women of childbearing age. MS has two principal forms, which are relapsing-remitting multiple sclerosis (RRMS) being more common and primary progressive MS (PPMS) [2]. More clinical forms, including secondary progressive MS and clinically isolated syndrome, have also been described [3].

Although the process of neurodegeneration in MS is still not well-understood, the role of immunological pathways and subsequent inflammation has been well-established and has dramatically impacted the development of highly effective therapies for MS. In MS, peripheral regulation is deficient either due to decreased regulatory T cells (Treg) and/or increased resistance of effector T and B cells to the suppressive mechanism [4,5]. Activated leukocytes from MS patients can enhance blood-brain barrier (BBB) permeability by expressing and secreting inflammatory cytokines, reactive oxygen species, and matrix metalloproteinases that disrupt the tight junctions and alter the basement membrane proteins of the cells lining the BBB. This leads to increased permeability and leukocyte migration across the BBB into the central nervous system (CNS), which in turn leads to lesion development in MS [6]. Inflammation is a hallmark in the pathogenesis of the disease, more pronounced inflammation is observed in the acute phases. Early MS lesions show invading peripheral immune cells and leakage of the blood-brain barrier with macrophages dominating the infiltrate, followed by cluster of differentiation (CD)8+ T cells, whereas lower numbers of CD4+ T cells, B cells, and plasma cells can also be found. Eventually, the inflammation becomes organized inside the central nervous system, with fewer invading cells observed in the lesions during progression [5,6].

B cells can regulate various immune functions mediated by B and T cells. Some B cells play a pro-inflammatory role by secreting tumor necrosis factor-alpha (TNF-α) and lymphotoxin. In contrast, a different interleukin (IL)-10-producing B cell population has a regulatory, anti-inflammatory role. Interestingly, B cells in MS may have an inherent pro-inflammatory functional phenotype. B cells have many different roles in multiple sclerosis pathogenesis; they can differentiate into plasma cells and secrete autoantibodies that contribute to central nervous system (CNS) inflammation via opsonization of CNS antigens and complement fixation, can recognize and internalize the CNS antigens and act as antigen-presenting cells (APCs) to activate CNS‐specific pathogenic T cells. Different subsets of B cells in the CNS can modulate T‐cell and myeloid cell functions by secretion of pro‐inflammatory and anti‐inflammatory cytokines [6,7].

CD20 is a non-glycosylated phosphoprotein expressed on most B cells and a key mediator of B cell differentiation. With the advancement in medicine and revolutionary drug therapies, anti-CD20 monoclonal antibodies (mAbs) were developed which exert their function by targeting and binding to CD20. This binding leads to apoptosis by activating complements that will lead to complement-mediated cytotoxicity (CDC), antibody-dependent cell-mediated cytotoxicity (ADCC), or by direct cellular killing through crossing linking of multiple mAbs and thus causes B cell depletion.

In 2018, ocrelizumab was the first treatment to be approved for the primary progressive form of MS with inflammatory activity in Europe and France rituximab (RTX) is an IgG1 mouse-human monoclonal antibody against CD20 and was the first monoclonal anti-CD20 antibody to be licensed for use in human disease [8]. It is widely used as an off-label anti-CD20 therapy for MS. Ocrelizumab (Ocrevus®) is a humanized anti-CD20 mAb and was approved as a disease-modifying therapy (DMT) for PPMS and RRMS in Europe [9]. Ofatumumab is an anti-CD20 human monoclonal IgG1 antibody binding firmly to a distinct membrane epitope similar to rituximab and ocrelizumab and is the first type 1 immunoglobulin G1 kappa (IgG1κ) fully human monoclonal antibody. In August 2020, the FDA-approved ofatumumab as a therapy for all forms of relapsing MS, including CIS, secondary progressive MS, and RRMS. The study was done on November 30, 2020, and was under evaluation by the EMA, which was approved later on March 2021 [9]. There has been a vast increase in the number of disease-modifying therapy (DMT) for MS notably for the disease’s relapsing form. Their short-acting function is to prevent clinical relapses and disease activity on MRI, and the long-term objective is to stop the progression of disability from worsening. With a wide range of mechanisms of action, side effects, mode of administration, and dosing interval, the choice of a DMT is tailored to the patient’s clinical scenario. DMT is divided into first- and second-line treatment groups on the foundation of therapeutic escalation strategy, which refers to the initiation of a safe but generally less effective therapy to reduce long-term safety concerns. Patients transition over time to more potent medications as needed. This observational single-center registry-based study aims to evaluate the effect of anti-CD20 treatment for MS (RR and progressive) in a population-based cohort treated at the university hospital.

## Materials and methods

This is a retrospective, longitudinal observational study of anti-CD20 treatments available for utilization in definitive MS and its different clinical forms at a university hospital with a capacity of 105,354 patients. The approval of Comité Local d’Ethique de la Recherche en Santé (CLERS) was obtained on February 24, 2021. The participants of our study were from a large cohort of 35,000 patients with MS registered under the French organization Observatoire Français de la Sclérose en Plaques (OFSEP). Patient data for this cohort can be accessed and utilized through the European Database of Multiple Sclerosis (EDMUS), a software database developed for all patients suffering from MS in France. The patients in this study are followed up by neurologists in the university hospital, and their clinical data were extracted from the EDMUS software. Hospital medical records were used in case of discrepancies. All patients signed a consent form authorizing access and permission to utilize and capture their data in OFSEP. Patients diagnosed with MS according to McDonald’s diagnostic criteria 2010 aged 18 years and above were included in the study [10]. MS patients who were being treated with anti-CD20 at least once were included only in the comparative analysis whereas all patients receiving the treatment regardless of the duration, were incorporated only into the descriptive analysis. Data extraction was done on November 30, 2020. Patients with neuromyelitis optica spectrum disorder (NMOSD) and other inflammatory neurological diseases were excluded. Furthermore, any modifications or new patients added after November 30, 2020, were not included. One of the clinical parameters that were assessed in our study was MS relapses which are defined as the new occurrence or marked worsening of symptoms typical for inflammatory demyelinating disease of the CNS. The symptoms should not be due to fever or infection, and the duration of symptoms must be at least 24 hours. The number of relapses in 12 months preceding the first injection of anti-CD20 and the number of relapses in 12 months after the anti-CD20 injections were calculated. Each episode of relapse was verified and registered into the database by a neurologist specializing in MS. The second clinical parameter that was evaluated was the Expanded Disability Status Scale (EDSS), which is a score to monitor the disease’s clinical progression and the effectiveness of clinical interventions, e.g., treatments. A physician calculates EDSS at each medical visit or during the hospitalization in neurology daycare. These data are verified by two neurologists who specialized in MS before being registered into EDMUS. We collected the EDSS scores just before receiving the first dose of anti-CD20 (day 1), and the scores were calculated 12 months after the first dose. For radiological parameters, Barkhof’s radiological criteria for MS were used which are as follows: (a) at least one gadolinium-enhancing lesion or at least nine lesions on transverse relaxation time (T2)-weighted images, (b) at least three periventricular lesions, (c) at least one juxtacortical lesion, and (d) at least one juxtacortical lesion [11]. The number of lesions (T2-weighted periventricular, juxtacortical, and infratentorial cerebral lesions) in the cerebral MRI were counted by at least two neurologists specialized in MS before being entered into the database. The cerebral MRI sequence with gadolinium injection and medullary MRI was not included in the study as they are not routinely performed. We collected data on MRI at the start of the treatment and the number of lesions from the MRIs available, after an interval of 12 months. Serum IgG levels need to be assessed before each injection of anti-CD20 as low levels of IgG (hypogammaglobulinemia) were associated with a higher infection rate. To study the effects of IgG after anti-CD20, we collected the IgG level of patients before the first injection and were followed up for 12 months. The demographic variables that were being evaluated were age, sex, and history of smoking. The diagnostic factors being assessed were forms of MS, age at first relapse, whereas therapeutic factors were age at first DMT, age at starting anti-CD20, last DMT used before starting anti-CD20, reason to switch to anti-CD20, duration of anti-CD20 treatment. The primary outcomes measures being studied were the number of relapses during 12 months prior to starting anti-CD mAbs and the number of relapses 12 months after the treatment, and EDSS score at the start of the treatment (day 1) compared to EDSS score at one year of treatment. The secondary outcomes measures being assessed number of T2 lesions (periventricular, juxtacortical, and infratentorial non-medullary) at the start of treatment and after 12 months under treatment and IgG level at the beginning of the treatment and IgG level at 12 months of treatment.

Statisticial analaysis

**Figure 1:**
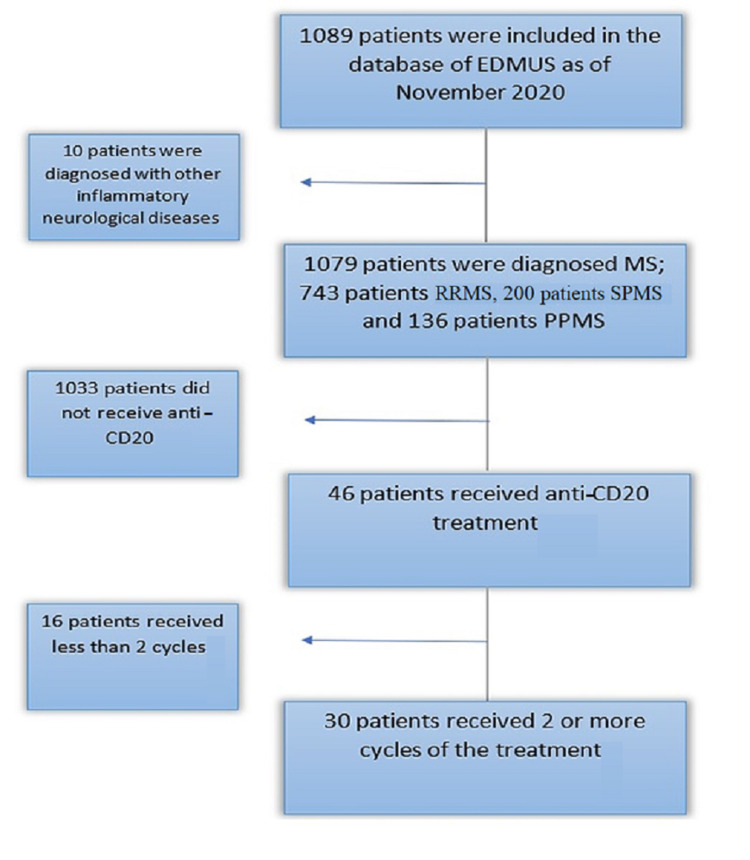
Patient's selection according to the database at university hospital in Normandy EDMUS: European Database of Multiple Sclerosis; MS: multiple sclerosis; RRMS: relapsing-remitting multiple sclerosis; CD: cluster of differentiation; SPMS: secondary progressive multiple sclerosis; PPMS: primary progressive multiple sclerosis

The descriptive analysis was done for the entire cohort, including all patients treated with anti-CD20 regardless of the duration of treatment (n =46) whereas all patients who were treated for less than 12 months were excluded. The data of the new cohort was analyzed using the R software version 4.0.2 (Vienna, Austria: The R Foundation) (Figure 1) [9,12]. Categorical variables were represented by frequencies and percentages, chi-square test (or Fisher’s exact test, as appropriate) was used to test the differences among the different types of MS. For continuous variables, means and standard deviations were represented using Skewness-Kurtosis tests to evaluate the normal distribution of the variables. Based on normality status, the one-way ANOVA or the Kruskal-Wallis test was used to differentiate patients with the different types of MS. Similarly, the paired t-test or the Wilcoxon signed-rank test compares findings before and after the anti-CD20 treatment.

## Results

Descriptive analysis of the entire cohort (n=46)

The study included a total of 46 patients, and 54.3% of patients were female. The average age of the participants was 43.9 ±9.4, and 28.3% of them were smokers (Table 1). Relapsing-remitting MS was the most common form with 23 patients, 14 patients were primary progressive and nine were secondary progressive.

**Table 1:** Baseline characteristics of the included patients (N=46) N: numbers; SD: standard deviation

Variables		Types of multiple sclerosis			
		Primary progressive	Relapsing-remitting	Secondary progressive	Total
Age in years; mean (SD)		45.2 (7.4)	40.4 (9.0)	50.7 (9.6)	43.9 (9.4)
Gender	Female	5 (35.7%)	15 (65.2%)	5 (55.6%)	25 (54.3%)
	Male	9 (64.3%)	8 (34.8%)	4 (44.4%)	21 (45.7%)
Smoking	No	10 (71.4%)	18 (78.3%)	5 (55.6%)	33 (71.7%)
	Yes	4 (28.6%)	5 (21.7%)	4 (44.4%)	13 (28.3%)

The average age of starting anti-CD20 was 42.9 ±8 years for the primary progressive (PP) form, 39.1±8.9 for the relapsing-remitting (RR), and 49.1±9.2 years for secondary progressive multiple sclerosis (SPMS). The mean delay from the first relapse to starting anti-CD20 was 117.9±94.7 months across all types of MS, 75.1±51.9 months for PPMS, 118±93.8 months for RRMS, and 184±117.2 months for SPMS (Table 2).

**Table 2:** Treatment history of included patients (N=46) **Five patients had abnormal LFTs, two patients had severe GI intolerance, and one patient had one of the following side effects (abnormal TSH, basal cell carcinoma, muscle pain, skin warts, hair loss, and lymphopenia). SD: standard deviation; GI: gastrointestinal; DMF: dimethyl fumarate; TSH: thyroid-stimulating hormone; LFT: liver function tests; DMT: disease-modifying therapy; CD: cluster of differentiation

Variables	Types of multiple sclerosis			
	Primary progressive	Relapsing-remitting	Secondary progressive	Total
Age at first relapse; mean (SD)	36.6 (±8.6)	29.3 (±7.2)	33.8 (±11.1)	32.4 (±8.9)
Age at first treatment; mean (SD)	39.1 (±9)	30.3 (±7.5)	40.3 (±12.5)	35.1 (±10.1)
Age to start anti-CD20; mean (SD)	42.9 (±8)	39.1 (±8.9)	49.1 (±9.2)	42.2 (±9.3)
Delay between first relapse and starting anti-CD20 (months); mean (SD)	75.1 (±51.9)	118 (±93.8)	184 (±117.2)	117.9 (±94.7)
Duration of anti-CD20 treatment (months); mean (SD)	18.9 (±7.4)	15.7 (±9.4)	23.3 (±12.5)	18.1 (±9.8)
Last treatment before anti-CD20				
Anti-CD20 was the first treatment	3 (21.4%)	3 (13%)	0	6 (13%)
Teriflunomide	1 (7.1%)	7 (30.4%)	0	8 (17.4%)
Biotin	5 (35.7%)	0	2 (22.2%)	7 (15.2%)
Glatiramer acetate	1 (7.1%)	2(8.7%)	1 (1.11%)	4 (8.7%)
Cyclophosphamide	1 (7.1%)	0	1 (1.11%)	2 (4.3%)
Fingolimod	0	7 (30.4%)	3 (33.3%)	10 (21.7%)
Mitoxantrone	1 (7.1%)	0	0	1 (2.2%)
Methylprednisolone	1 (7.1%)	0	0	1 (2.2%)
DMF	0	3 (13%)	0	3 (6.5%)
Natalizumab	0	1 (4.3%)	0	1 (2.2%)
No data	1 (7.1%)	0	2 (22.2%)	3 (6.5%)
Reason to change to anti-CD20				
Disease activity	8 (57.1%)	10 (43.5%)	5(55.6%)	23 (50%)
First treatment or no data	4 (28.6%)	3(13%)	2(22.2%)	9 (19.6%)
DMT intolerance*	2 (14.3%)	10 (43.5%)	2(22.2%)	14 (30.4%)

Disease activity (inflammatory activity including new relapses clinical and/or radiological changes) was the most common cause for changing DMT into anti-CD20 (50%), followed by DMT intolerance (30.4%), including abnormal liver enzymes (35.7%) followed by severe GI symptoms (14.3%). The average EDSS at the start of the treatment was 4.3±2 for all types of MS, 5.6±1 for PPMS, 2.3±1.3 for RRMS, and 5.9±1 for the SPMS. Regarding the treatment history, the most common treatment received before anti-CD20 was fingolimod (n=10), followed by teriflunomide (n=8), and biotin (n=7), respectively. Anti-CD20 was the first treatment for six patients. Therefore, a total of 14 patients (30.4%) received second-line treatment (immunosuppressor) before starting anti-CD20, 15 patients (32.6%) had a first-line treatment (immunomodulators), and six patients (13%) had anti-CD20 as a first-line treatment. Fourteen patients received one DMT before starting anti-CD20, nine patients received two DMTs, six patients did not receive any DMT, six patients received four DMTs, five patients received five DMTs, four patients received three DMTs, and two patients received eight DMTs.

Descriptive and comparative analysis of the refined cohort (n=30)

The secondary analysis included 30 patients with a mean age of 44.2±10.1 years. About 57% of the included patients were males, and around 27% of them were smokers. Regarding the types of MS, 12 patients had PPMS, 12 patients had RRMS, and six had SPMS. There was a significant difference among patients’ age compared to different types of MS (P=0.044). However, there were no significant differences in terms of gender or smoking habit. The anti-CD20 was the primary treatment in seven patients, while biotin was the most common treatment used before anti-CD20, followed by teriflunomide (n=5), fingolimod (n=4), and glatiramer acetate (n=3). The mean duration from the first relapse to adopting the anti-CD20 treatment was 122.0±102.6 months. The mean duration of the anti-CD20 treatment was 22.6.0±9.0 months, with an average of 4.1±1.2 treatment cycles. Disease activity was the most common cause (56.7%) of switching into the anti-CD20 treatment, followed by DMT intolerance, including severe gastrointestinal intolerance (6.7%). Regarding the effect of the anti-CD20 treatment on the disease course, there was a significant reduction (P=0.001) in the number of relapses in the last 12 months following the start of the anti-CD20 (0.1±0.3) as compared to the number of relapses prior to anti-CD20 (0.4±0.5). However, there was a significant reduction in the IgG levels (P=0.017) when comparing its values before (9.8±2.5 g/L) and after (9.2±2.4 g/L) starting the anti-CD20 treatment. Nevertheless, there was no significant change (P=0.952) in the Expanded Disability Status Scale after the anti-CD20 treatment as compared to before it (Table 3).

**Table 3:** Changes in clinical parameters following anti-CD20 treatment *Statistically significant. N: numbers; CD: cluster of differentiation

Variables	Before anti-CD20 treatment	After anti-CD20 treatment	P-value
	Mean ± SD	Mean ± SD	
Number of relapses in 12 months (N=30)	0.4 ± 0.5	0.1 ± 0.3	0.001*
Expanded Disability Status Scale (N=30)	4.4 ± 2	4.4 ± 2.3	0.952
Immunoglobulin G Levels (g/L) (N=27)	9.8 ± 2.5	9.2 ± 2.4	0.017*

The MRI findings were available for 13 patients only, which may explain the absence of statistical significance when compared with the results of the first MRI at the start of the anti-CD20 to the MRI one year later (Table 4). Across different MS types, we found a significant reduction in the number of relapses in the RRMS while stability in the PPMS and SPMS. Concerning the EDSS, we found a slight increase in the PPMS while a relatively stable EDSS Score in RRMS, whereas a slight decrease in the SPMS was observed (Table 5).

**Table 4:** Changes in MRI findings following anti-CD20 treatment (N=13) MRI: magnetic resonance imaging; SD: standard deviation; CD: cluster of differentiation

Variables	First MRI after treatment	MRI in 1 year of treatment	P-value
	Mean ± SD	Mean ± SD	
Number of T2 periventricular lesions	10.1 ± 6.0	10.1 ± 6.0	1.000
T2 juxtacortical lesions	6.9 ± 5.1	7.0 ± 5.1	0.317
T2 infratentorial non-medullary lesions	1.4 ± 2.7	1.4 ± 2.7	1.000

**Table 5:** Changes in clinical parameters following anti-CD20 treatment according to type of MS SD: standard deviation; CD: cluster of differentiation; EDSS: Expanded Disability Status Scale; MS: multiple sclerosis

		Relapses in 12 months before treatment	Relapses in 12 months after treatment	EDSS at the start of treatment	EDSS after 12 months of treatment
Type of multiple sclerosis	Primary progressive (mean ± SD)	0.1 ± 0.3	0.1 ± 0.3	5.6 ± 1	5.9 ± 1.2
	Relapsing-remitting (mean ± SD)	0.7 ± 0.5	0.1 ± 0.3	2.3 ± 1.3	2.2 ± 1.5
	Secondary progressive (mean ± SD)	0.7 ± 0.5	0	5.9 ± 1	5.7 ± 1.6

## Discussion

In our study, the average age of diagnosis for MS was 32.5±8.9 years, which is similar to an observational study by Vukusic et al. in 2020 including 68,097 patients reporting the average age of diagnosis as 32.5±10.7 years [13]. The average age of onset was also identical to the Swedish study by Salzer et al. showing an average of 31.3±10.5 years [14]. The male to female ratio in this study shows a female predominance in the RR form and a male predominance in the PP form, whereas a relatively equal sex ratio in SPMS. The systematic review by Alonso and Hernán found similar results in RRMS with a female predominance [15]. However, the results of our study regarding gender predisposition in PPMS varied when compared to the study of Tremlett et al., which described the natural history of PPMS in a subgroup of 2837 patients from British Columbia, Canada, and showed an equal male to female ratio of 1:1 [16]. The male predominance in PPMS observed in our study can be explained by the small sample size, which is non-representative of the general population. Furthermore, the results might be affected by a selection bias, as neurologists often prescribe anti-CD20 for the rapidly progressive and more severe form of the disease, which is usually seen in males according to the study done by Confavreux and Vukusic published in 2014 [2]. Nevertheless, according to our study and in literature, it is evident that the ratio of affected males is higher in PPMS than the ratio of males affected in RRMS (1:1 vs. 2:1). This increase in the ratio might be due to apprehension and attitude of delay in seeking help observed in males as hypothesized by Eccles in 2019 [17]. The overall male to female ratio across all types of MS in this study was 0.85:1, which is different from the ratio observed in the observational study by Vukusic et al. that showed a ratio of 0.4:1. This difference might be due to sample size and the high percentage of PPMS in our study [13].

The average delay from onset to starting anti-CD20 is 9.8±7.8 years for RRMS, 6.3±4.3 years for PPMS, and 15.3±7.9 years for SPMS. However, a comparison of our results to other studies revealed different results. For RRMS, a shorter delay of only 4.15±4.95 years was observed in the OPERA II trial [18]. For PPMS, we found a much shorter delay in the ORATORIO study (2.9±3.2 years) [13], while the Swedish study showed a longer delay of 8.2±6.2 years [14]. In our study, the delay in the SPMS was principally shorter than the delay shown in the Swedish cohort by Salzer et al. in 2016 (19.1±7.9) [14]. The shorter delay observed in the OPERA trial and ORATORIO might be due to their eligibility criteria. Patients with a disease duration of fewer than 10 years were not included in the OPERA trial, similarly, patients with a duration of fewer than 15 years were not included in the ORATORIO trial. In general, the longer delay for starting anti-CD20 after disease onset observed in SP and PP is logical since the diagnosis of these progressive forms requires a retrospective or prospective follow-up for at least one year before defining the disease’s clinical pattern and confirming the diagnosis.

The average EDSS score at the start of anti-CD20 for this study is 2.3±1.3 for RRMS, 5.6±1 for PPMS, and 5.9±1 for the SPMS. The Swedish study by Salzer et al. in 2016 reports similar findings: for RRMS with an average EDSS score of 2, PPMS with an average EDSS of 5, and SPMS with an average EDSS of 5.5 [14]. The disability progression in patients measured by EDSS scores shown in the PP forms compared to the RR form of the disease is most likely related to the delay in a confirmed diagnosis, the progressive non-remitting nature of these types, and the absence of approved efficacious treatment for a long time until the approval of ocrelizumab in 2011 [19]. With regards to physical disability based on EDSS scores, disability progression was becoming stable in RRMS after anti-CD20 (2.3 vs. 2.2 after 12 months), there is a slight increase in the PPMS, and a small reduction is observed in the SPMS (5.9 vs. 5.7). In the OPERA trials, the number of patients with a confirmed progression of disability after 12 weeks of treatment was significantly lower with ocrelizumab than with interferon beta-1a (9.1% vs. 13.6%), which is different from what has been observed in our study; it might be due to different methodology and different duration of EDSS follow up (12 weeks vs 12 months). The ORATORIO trial for PPMS showed a slight reduction in the percentage of patients with 12 weeks confirmed disability progression (32.9% with ocrelizumab vs. 39.3% with placebo) [13]. Our study results were similar to the results in the observational study by Salzer et al. [14]. The EDSS after 14.5 months of starting the treatment showed that the median EDSS remained unchanged in patients with RRMS (50.42) and increased by 0.5 and 1.0 for patients with SPMS and PPMS, respectively. The difference between the results observed in our study and the result shown in the ORATORIO trial is likely due to the difference in methodology and eligibility criteria (younger patients and shorter delay for introduction of treatment). The persistent progression in disability in PPMS observed in our study and the Swedish study might be attributed to the absence of a neuroprotective role of these treatments. With regards to the disability progression, we found relative stability in the EDSS with RRMS (2.3 vs. 2.2 after 12 months), a slight increase in the PPMS, and a small reduction is observed in the SPMS (5.9 vs. 5.7).

In this study, we found a statistically significant reduction of the number of relapses during the 12 months following the treatment across all MS types. The most notable reduction was in the RRMS with a decrease of 0.6 and SPMS. No reduction was observed in the PPMS form. In the OPERA trials, it shows a reduction of annualized relapse rate (ARR) with ocrelizumab compared to interferon beta-1a (0.16 vs. 0.29). In the Swedish observational study by Salzer et al., the ARRs were 0.044 in RRMS for patients under rituximab, 0.015 for PPMS, and 0.038 for SPMS [14]. The multicenter, randomized, double-blind, placebo-controlled study by Kappos et al. showed a reduction of ARR by 80% (95% CI: 45-99) in the ocrelizumab group comparing it to placebo [19]. Lastly, the HERMES trial by Hauser et al. showed a reduced number of relapses in the rituximab group in comparison to the placebo group at week 24 (14.5% vs. 34.3%, P=0.02) and week 48 (20.3% vs. 40.0%, P=0.04) [18]. The significant reduction in relapses shown in our study and the studies mentioned above is an indication of the effective anti-inflammatory action of these medications in MS, notably in the RRMS. The absence of reduction of relapses in PPMS noted in our study is probably related to its pathogenesis and the clinical characteristic of this form (non-relapsing MS).

We found no changes in the number of lesions in the cerebral MRIs of the 17 patients in this study. In the OPERA trials, the mean number of gadolinium-enhancing lesions per T1-weighted magnetic resonance scan was 0.02 with ocrelizumab versus 0.29 with interferon beta-1a in trial 1 and 0.02 versus 0.42 in trial 2 (95% lower number of lesions, P<0.001). In the ORATORIO trial, the total volume of brain lesions on T2-weighted MRI decreased by 3.4% with ocrelizumab and increased by 7.4% with placebo. The study by Kappos et al. reported a highly significant difference in ocrelizumab groups (P<0.0001) for a total number of gadolinium-enhancing T1 lesions at weeks 12, 16, 20, and 24 versus placebo [19]. Lastly, the HERMES trial by Hauser et al. showed a reduced count of total gadolinium-enhancing lesions at weeks 12, 16, 20, and 24 (P<0.001) and of total new gadolinium-enhancing lesions over the same period (P<0.001) for patients receiving rituximab, these results were sustained for 48 weeks (P<0.001) [18]. The above results are incomparable to our study due to the small sample size and the absence of systematic injection of gadolinium in follow-up MRIs.

Hypogammaglobulinemia is defined as immunoglobulins (Ig) less than 6g/L wherein normal values are between 6 and 16 g/L. Hypogammaglobulinemia is a well-described long-term adverse effect of rituximab, occurring in up to 50% of patients treated for hematological or rheumatological disorders [20]. In this study, we found a statistically significant reduction in serum immunoglobulin G (IgG) value after 12 months from the start of treatment (P=0.017). Among the 26 patients studied, only one (3.8%) patient developed hypogammaglobulinemia with IgG less than 6 g/L. None developed hypogammaglobulinemia of less than 5 g/L. We found multiple studies that showed a reduction in the IgG levels after starting rituximab or ocrelizumab as a DMT. However, only a small number of patients developed a significant decrease of IgG with a value of less than 5 g/L. In a study by Bar-or et al., the temporal evolution of immunoglobulin levels and the risk of severe infections was evaluated by baseline IG quartile in the phase III OPERA I, OPERA II, and ORATORIO [21]. A mean change in IgG levels in the first year of treatment was reported to be -0.24 (-2.9%) in OPERA and -0.23 (-2.9%) in ORATORIO. The study by Vollmer et al. included 527 MS patients treated with rituximab and ocrelizumab. The results showed 7.4% of patients developed hypogammaglobulinemia at less than 5 g/L after a mean of 36.1 months following treatment [22]. Another study by Tran et al. in 2019 reported that out of 168 patients treated with ocrelizumab for one year, 8.5% of patients developed IgG <6 g/L [23]. Therefore, hypogammaglobulinemia is frequently observed in patients treated by anti-CD20, and between 5% and 7% of patients develop severe hypogammaglobulinemia (serum IgG less than 5g/L). The current French recommendation proposes discontinuation of anti-CD20 treatment if values of IG become equal to or less than 5g/L. However, these recommendations are subject to future research in order to achieve conclusive evidence.

Our study experienced several limitations. As this was a single-center study, only patients at the university hospital were included whereas patients consulted by a neurologist in different hospitals and clinics were omitted. This led to a small sample size and hence the comparative analysis for the number of relapses in the 12 months preceding and after the treatment was not compared against other DMTs or placebo. There was also lack of a standardized protocol for medullary and cerebral MRI. Due to the alternate administration of ocrelizumab and rituximab for a single patient, the study included both types of anti-CD20 mAbs in a single cohort. Although the mechanism of action for both treatments is similar, they are not the same treatment. Hence, the prescription of ocrelizumab in France prior to its approval was only allowed for some patients with PPMS as a therapeutic protocol for clinical trials or as the temporary authorization for use (temporary authorization to use {ATU}). Those patients were reverted to rituximab after the withdrawal of ATU or the end of the therapeutic protocols.

## Conclusions

Anti-CD20 antibodies (rituximab and ocrelizumab) as disease-modifying therapy at university hospital showed a significant reduction in the number of relapses observed after 12 months of treatment; it showed no changes in the disability progression measured by the EDSS score for the relapsing-remitting form of the disease, while it increased in the primary progressive form. This result might reflect the effective anti-inflammatory characteristic of anti-CD20 in RRMS and the lack of neuroprotective characteristics of these treatments in the PPMS. Since the approval of ocrelizumab for MS, increasingly more patients will benefit from this effective treatment at a younger age and at an earlier stage, which can positively impact the quality of life in patients living with multiple sclerosis.
